# Effect of Land Cover Fractions on Changes in Surface Urban Heat Islands Using Landsat Time-Series Images

**DOI:** 10.3390/ijerph16060971

**Published:** 2019-03-18

**Authors:** Tao Chen, Anchang Sun, Ruiqing Niu

**Affiliations:** 1 Institute of Geophysics and Geomatics, China University of Geosciences, Wuhan 430074, China; sacsyd2007@126.com (A.S.); rqniu@163.com (R.N.); 2 Beijing North-Star Digital Remote Sensing Technology Co.Ltd., Beijing 100120, China

**Keywords:** surface urban heat island, land surface temperature, Multiple Endmember Spectral Mixture Analysis, ternary triangle contour graphics, time-series images

## Abstract

Man-made materials now cover a dominant proportion of urban areas, and such conditions not only change the absorption of solar radiation, but also the allocation of the solar radiation and cause the surface urban heat island effect, which is considered a serious problem associated with the deterioration of urban environments. Although numerous studies have been performed on surface urban heat islands, only a few have focused on the effect of land cover changes on surface urban heat islands over a long time period. Using six Landsat image scenes of the Metropolitan Development Area of Wuhan, our experiment (1) applied a mapping method for normalized land surface temperatures with three land cover fractions, which were impervious surfaces, non-chlorophyllous vegetation and soil and vegetation fractions, and (2) performed a fitting analysis of fierce change areas in the surface urban heat island intensity based on a time trajectory. Thematic thermal maps were drawn to analyze the distribution of and variations in the surface urban heat island in the study area. A Multiple Endmember Spectral Mixture Analysis was used to extract the land cover fraction information. Then, six ternary triangle contour graphics were drawn based on the land surface temperature and land cover fraction information. A time trajectory was created to summarize the changing characteristics of the surface urban heat island intensity. A fitting analysis was conducted for areas showing fierce changes in the urban heat intensity. Our results revealed that impervious surfaces had the largest impacts on surface urban heat island intensity, followed by the non-chlorophyllous vegetation and soil fraction. Moreover, the results indicated that the vegetation fraction can alleviate the occurrence of surface urban heat islands. These results reveal the impact of the land cover fractions on surface urban heat islands. Urban expansion generates impervious artificial objects that replace pervious natural objects, which causes an increase in land surface temperature and results in a surface urban heat island.

## 1. Introduction

The process of urbanization transforms the natural landscape for anthropogenic urban land uses, which leads to changes in the physical characteristics of the surface of the transformed areas [1]. Urbanization is likely to cause both significant changes in land use and land cover and severe environmental deterioration. One of the environmental consequences of urbanization is the surface urban heat island (SUHI) effect. The SUHI effect was first proposed by Howard, who found relatively higher temperatures in urban areas than in the surrounding suburban/rural areas [2]. The SUHI effect reduces human comfort and is harmful to urban residents [3,4]. The SUHI effect has currently attracted worldwide attention and become one of the most important research areas in urban climate and urban environmental research [5].

The first SUHI observations that used remote sensing technology were reported by Rao [6], who found that remote sensing images can enable spatially continuous analyses compared with the sparse coverage obtained by meteorological monitoring of SUHI effects. Since then, a variety of sensor platforms using various methods have been applied. For example, Advanced Very High-Resolution Radiometer (AVHRR) and Moderate-Resolution Imaging Spectroradiometer (MODIS) data have been used to evaluate and map SUHI effects at a macro scale [7,8,9,10,11,12,13,14], whereas Thematic Mapper and Thermal Infrared Sensor (TM&TIRS) data have primarily been used to study the effects at the city level [15,16,17,18,19]. The higher resolution of aerial thermal data may generate more detailed temperature distribution information [20]. In recent years, researchers have focused on the effect of urbanization land use and land cover (LULC) on the SUHI, and their experiments have classified the research area and produced statistics on different land cover categories [21,22]. Moreover, some common indicators, for example, the normalized difference vegetation index (NDVI), have provided reference indicators to evaluate the SUHI with urban surface change [23,24]. Instead of the NDVI, researchers have found that spectral information can be obtained from the vegetation fraction, which makes the indicator more reliable [25,26,27,28]. Compared with these two coefficients, the percentage of impervious surface is more stable and less affected by seasonal change [29,30]. Previous studies have focused on urban impervious surfaces, which have a different relationship with the SUHI at different scales [31,32,33,34]. However, these studies have mostly focused on one or two remote sensing images. Long-term remote sensing data are important for monitoring SUHI growth [35] and can also provide more detailed information on the evolution of SUHIs as well as land cover change [36]. 

In this study, we explored the connection between the SUHIs and land cover fraction (LCF) in the Metropolitan Development Area of Wuhan by using six Landsat images acquired in September and October during the period of 1990 to 2014. We used the generalized single-channel method and the radiation transfer equation method to retrieve the land surface temperature (LST), and thematic thermal maps were then drawn to analyze the characteristics of the experimental areas. Additionally, we used the Multiple Endmember Spectral Mixture Analysis (MESMA) method to extract the land cover fraction information. Then, a mapping method was used to generalize the six ternary triangle contour graphics, which intuitively reflect the distribution and variation relationships between three land cover endmember fractions and normalized LST. Instead of performing the regression of the SUHIs with fractional information at a single time, we used time-series-normalized LST images to build a time trajectory of each pixel. Then, four features were summarized based on the time trajectory change characteristics. A specific feature was then extracted, which is defined as the SUHI large change area. A linear fitting was used to explore the relationship between the land cover fraction and SUHI intensity. 

The remainder of this study is organized as follows. Section 1 gives a brief introduction of the research area, research data and data preprocessing. Section 2 presents the LST retrieval method and MESMA. Section 3 shows the results of the experiment. Section 4 presents the discussion and Section 5 presents the concluding remarks.

## 2. Materials and Methods 

### 2.1. Study Area

**Figure 1:**
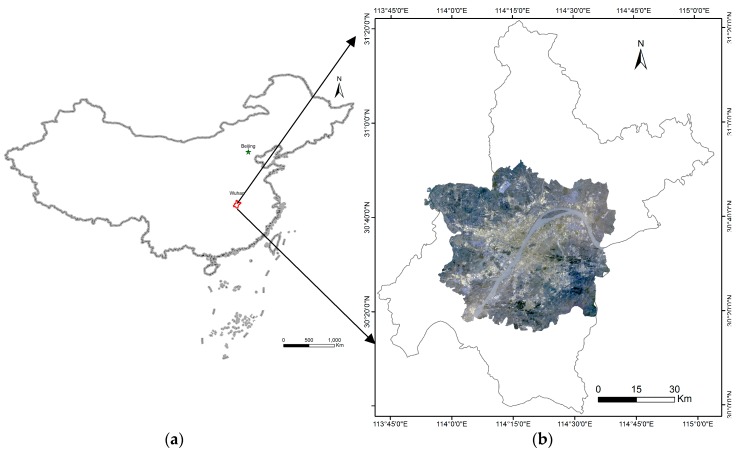
Location of study area (R: Band4, G: Band3, B: Band2 of Landsat 8 image).

The Metropolitan Development Area of Wuhan was chosen as the study area in this research. Wuhan, the capital of Hubei Province, is located at the intersection point of the Yangtze River and the Han River (113°41′E-115°05′E, 29°50′N-31°22′N) (Figure 1). Wuhan is a flat watery land crisscrossed by rivers and lakes, and it has a subtropical monsoon climate with four seasons, adequate illumination and abundant rainfall. Wuhan is regarded as one of the hottest “stove cities” in China. During the summer, the subtropical high system generally dominates the city of Wuhan. The rivers and lakes produce higher relative humidity, which leads to an uncomfortable apparent temperature. Additionally, according to the meteorological data, the Wuhan annual average temperature has greatly increased over the last 50 years. The extreme heat condition (>35 °C) of Wuhan occurs for 1.4–5.5 days longer on average than the surrounding county. 

Wuhan is also the most flourishing city in central China and has a population of 8.217 million. In 2011, the Wuhan government proposed the creation of the Metropolitan Development Area, which consists of one main urban area with six suburban areas and covers 3403.99 km^2^. The Metropolitan Development Area of Wuhan contains the urban district and defines a boundary for city development.

### 2.2. Data and Preprocessing

Five Landsat-5 images and one Landsat-8 image were chosen for this research. The Landsat-5 satellite has special advantages in obtaining long-term images because it has more than 30 years of available and free data with wide coverage. Landsat-5 images are also frequently used in SUHI experiments because they have appropriate resolutions: 30 m for multi-spectral bands and 120 m for the thermal infrared band. Additionally, we used Landsat-8 images of the last few years to supplement our dataset. All images were downloaded from the International Scientific and Technical Data Mirror Site (http://www.gscloud.cn). These image products were carefully selected based on three considerations: (1) The data should be in the same season, in the same month is the best; (2) the image should be good quality, with less cloud cover over the study area; (3) the image should have appropriate time intervals. Landsat images used in this study are shown in Table 1.

**Figure 2:**
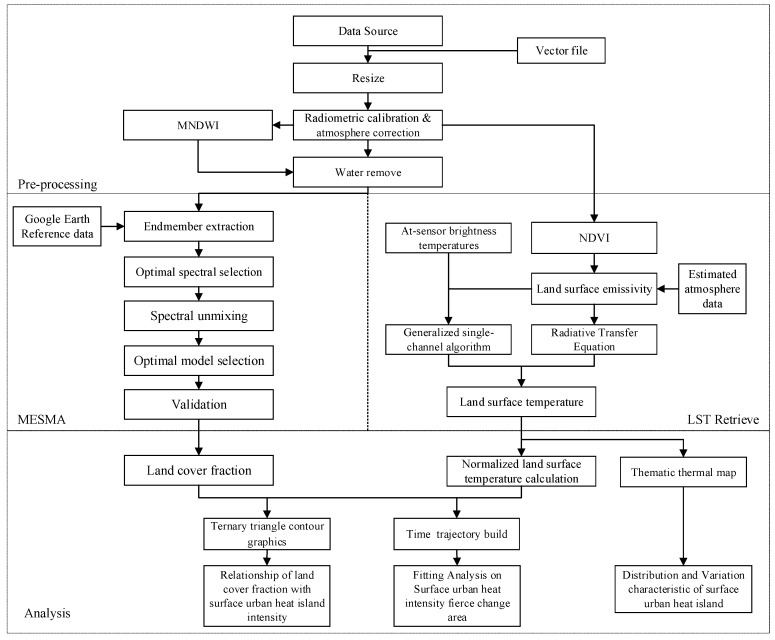
Flow chart for this study.

The downloaded images were all level 1T products that had already been corrected for radiation and geometry. First, a vector file was generated by vectorization to resize the six time images, including the multi-spectral and thermal infrared bands. Second, we resampled all the images to 60 m using the nearest-neighbor method because the downloaded Landsat data had been resampled to a 30-m resolution, which we thought was inappropriate for further analysis. Then, radiometric calibration and atmosphere corrections were performed to switch the DN (Digital Number) to reflection. Finally, water areas were removed from all images using the modified normalized difference water index (MNDWI). All steps were performed using the ENVI 5.0 and ArcGIS 10.0 software platforms. The entire flow chart for this study is shown in Figure 2. 

### 2.3. Method

#### 2.3.1. LST Retrieval

Two LST retrieval methods were used in this research: the generalized single-channel algorithm and the radiative transfer equation algorithm [37,38]. The generalized single-channel algorithm was used for Landsat 5 data because the algorithm’s parameters are not suitable for Landsat 8 data. The radiative transfer equation algorithm was used for Landsat 8 data. Although Landsat 8 has two bands (band 10 and band 11) in the thermal infrared band, band 10 is preferred because the information in band 11 has some uncertainty [39]. 

Landsat thermal infrared bands were first transferred to at-sensor brightness temperatures under the assumption of uniform emissivity [40]. Then, we used a modified retrieval surface emissivity method proposed by Cao [41] that is based on the NDVI threshold model [42] and has been shown to achieve more precise results. The atmospheric profile parameters were estimated from a NASA (National Aeronautics and Space Administration) website (http://atmcorr.gsfc.nasa.gov). Finally, the generalized single-channel algorithm and the radiative transfer equation algorithm were used to retrieve the LSTs. 

#### 2.3.2. Multiple Endmember Spectral Mixture Analysis (MESMA)

The MESMA method proposed by Roberts [43] was used in this research to extract fractional information. This unmixing method dynamically adjusts the number of endmembers when processing mixed pixels and then evaluates and selects the best endmember spectra from all possible combinations for each pixel [44]. MESMA has been used to map urban land cover at the sub-pixel level and provide persuasive results in describing urban material distributions [45,46,47,48,49]. Each step in MESMA is explained as follows.

First, suitable endmembers should be extracted to create the spectral libraries. We used the concept proposed by Ridd, who assumed that the spectral signature of land cover in urban environments is a linear combination of three components: vegetation, impervious surfaces and soil [50]. In this study, we defined three categories of endmembers: (1) impervious surfaces (IS), which contain all man-made impervious materials such as roofs and roads; (2) green vegetation (VEG), which contains all chlorophyllous vegetation; (3) non-chlorophyllous vegetation and soil (N&S), which contains bare land, soil and some vegetation without chlorophyll. 

Endmember extraction was individually performed for certain Google Earth images. Then, six original spectral libraries were built. Optimal endmember spectra were chosen from the libraries using three indices: Endmember Average RMSE (root-mean-square error) (EAR) [51], Minimum Average Spectral Angle (MASA) [52], and Count-Based Endmember Selection (CoB) [53]. We chose the relatively high CoB spectra, although the EAR and MASA indices were considered when the CoB index was similar. The lowest MASA spectra were chosen for dark objects, such as certain impervious surfaces, and the lowest EAR spectra were chosen for bright objects, such as certain vegetation. 

The optimal spectral libraries were used in the spectral unmixing. Photometric shade (SHD) was added as the endmember to account for variations in illumination. Two-, three-, and four-endmember models were applied in this study. According to previous studies [48,49,50,53], the fractions of 1.05 (maximum) and −0.05 (minimum) were used as constraints. An additional range of 5% was incorporated to consider noise-oriented modeling errors [47]. The RMSE of each pixel was constrained to less than 0.025. 

The two-, three-, and four-endmember unmixing models were selected to reach the lowest RMSE. Generally, the RMSE in a pixel decreased when a greater number of endmembers was used in the MESMA. Therefore, two threshold values were set based on previous research [53]. Specifically, the reflectance threshold value between the lowest RMSE two- and three-endmember models was 0.7% and that between the three- and four-endmember models was 0.2%.

Because of the lack of surface fractional data, the experiment could not provide an accurate evaluation from the fractional level. Therefore, the experiment assumed that if the fraction of an endmember was greater than 0.5, the pixel should be dominant in the hard classification. Finally, 100 pixels for each endmember were extracted. The overall accuracy and Kappa coefficients were used to validate the MESMA.

#### 2.3.3. SUHI Evaluation Factors

After LST retrieval, a robust statistical method was used to produce the thematic thermal map [41]. We divided the LST into seven regions: extreme high, high, sub-high, medium, sub-low, low and extreme low. Each region is defined in Table 2, where LST mean is the average LST, and STD is the standard deviation.

Additionally, a normalized LST value was defined as follows:

(1) $${\mathrm{LST}}_{\mathrm{normalize}}=\frac{\mathrm{LST}-{\mathrm{LST}}_{\min}}{{\mathrm{LST}}_{\max}-{\mathrm{LST}}_{\min}}$$

where LST is the surface temperature in each pixel and LST_max and LST_min are the maximum and minimum surface temperatures, respectively. The normalized LST can reflect the relative temperature regardless of uncorrelated factors. In this experiment, we considered that high normalized LSTs reflected high intensity levels of the SUHI.

## 3. Results

### 3.1. LCF Results and Validation

**Figure 3:**
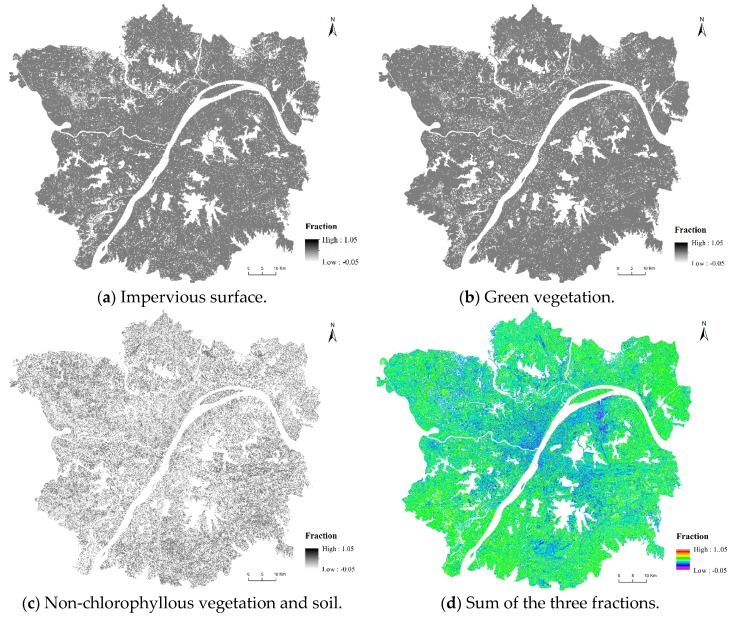
The land cover fraction (LCF) maps of 2014.

Figure 3 is an example of the LCF results based on MESMA. The area variation of the three endmembers during 1990 to 2014 is shown in Table 3.

From Table 3, it can be seen that the area of the IS endmember increases from 1990 to 1996, then decreases from 1996 to 2002. In 2009, the area of the IS endmember shows a slight increase, then decreases from 186.04 km^2^ in 2009 to 128.42 km^2^ in 2014. 

From the point view of the area of the VEG endmember, it decreases from 395.3 km^2^ in 1990 to 310.95 km^2^ in 1996, then the area has a slight increase in 2002 with an area of 360.01 km^2^. However, in 2009, the area declines to 319.95 km^2^. Then in 2014, the area increases from 319.95 km^2^ to 379.24 km^2^.

In terms of area of the N&S endmember, it shows a tendency similar to that of the IS endmember. The area of the N&S endmember first increases from 1990 to 1996, then it has a significant decline from 82.66 km^2^ (1996) to 18.76 km^2^ (2002). It increases significantly from 18.76 km^2^ in 2002 to 109.01 km^2^ in 2009. Finally, in 2014, it decreases to 75.69 km^2^.

The highest accuracy in Table 4 occurred in 1990, with an overall accuracy of 87.56% and a Kappa coefficient of 0.81. The worst results occurred in 2014, with an overall accuracy of 75.19% and a Kappa coefficient of 0.63. The results show that the pixel decomposition was successful and can be applied in further studies [47,49].

### 3.2. Distribution Characteristic of SUHIs

**Figure 4:**
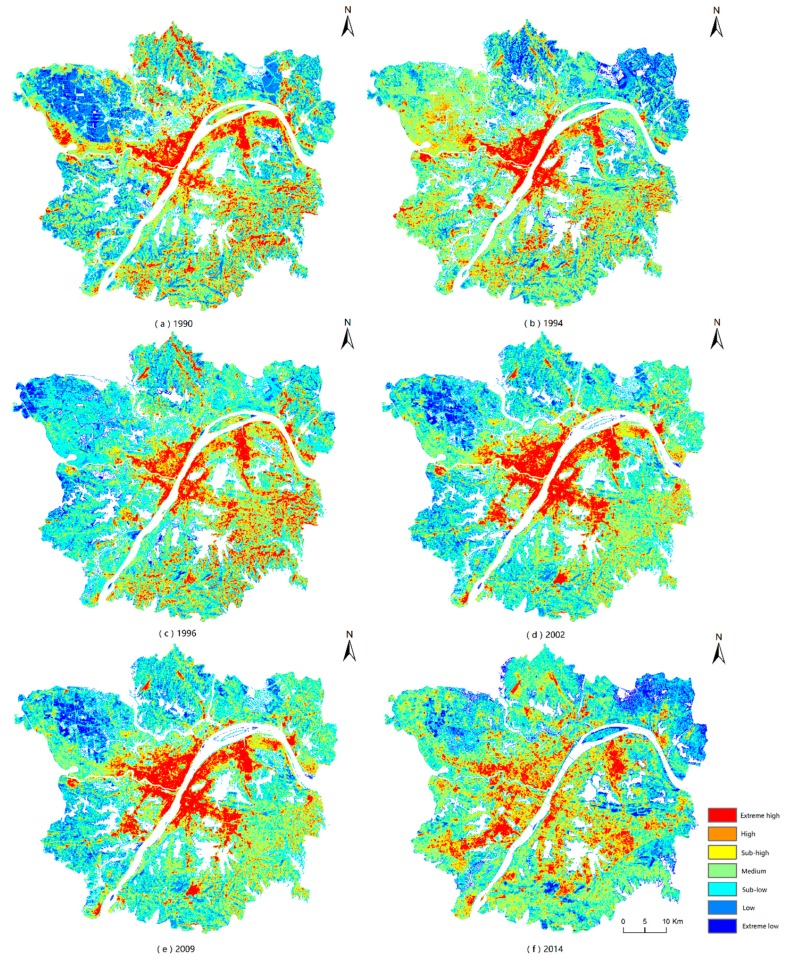
The thematic thermal map of the experimental area.

The thematic thermal maps are shown in Figure 4. The figure reveals the LST trends from low to high as well as the hue trends from cold to warm. The extreme high region was mostly located in the center of the city. The extreme high regions may relate to the urban area. The high and sub-high regions surrounded the extreme high region as a buffer zone. The extreme low regions were in specific areas, mostly in the left and right top corners of the image. Additionally, the low and sub-low region represented a transition from cold to warm.

**Figure 5:**
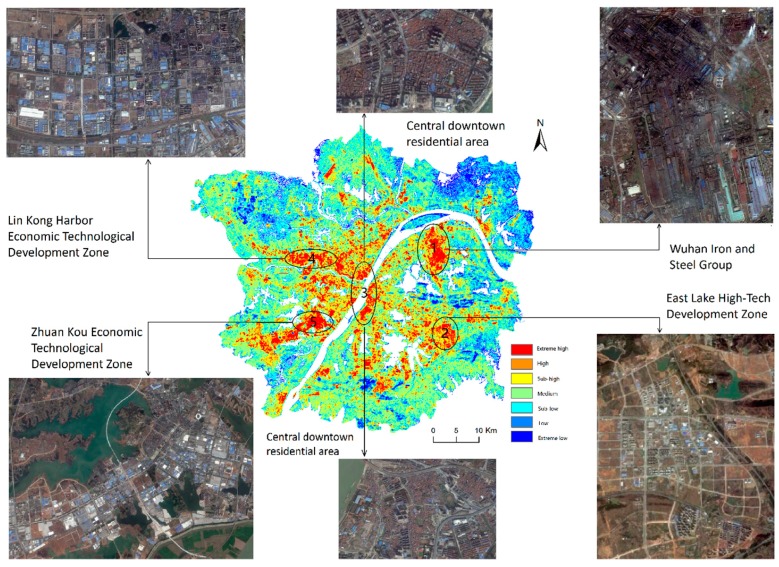
The aggregation sketch map of Wuhan SUHIs (based on the 2014 image).

The experiment focused on the image of 2014. Five aggregations of SUHIs are summarized and shown in Figure 5. The five areas circled in Figure 5 are the aggregations of SUHIs in Wuhan. According to the Google map data, the five areas are the Wuhan Iron and Steel Group, East Lake High-Tech Development Zone, Lin Kong Harbor Economic Technological Development Zone, Zhuankou Economic Technological Development Zone and central downtown residential area.

Circle 1 is the area of the Wuhan Iron and Steel Group, which may represent the hottest location in the Wuhan area. This area is covered with crowded buildings, mostly industrial plants and chimneys. Circles 2, 4 and 5 are three economic technological development zones that also belong to the aggregation of the extreme high regions. The boundaries of high and low temperatures are very clear. The East Lake High-Tech Development Zone is still under construction, whereas the other two have already been built. Circle 3 is the central downtown residential area, which belongs to the old town of Wuhan, and it is covered with high-density but short red tile-roofed houses. The other surrounding residential or commercial areas in the old town have relatively low aggregations of extreme high regions compared with that of Circle 3.

### 3.3. Variation Characteristics of SUHIs

**Figure 6:**
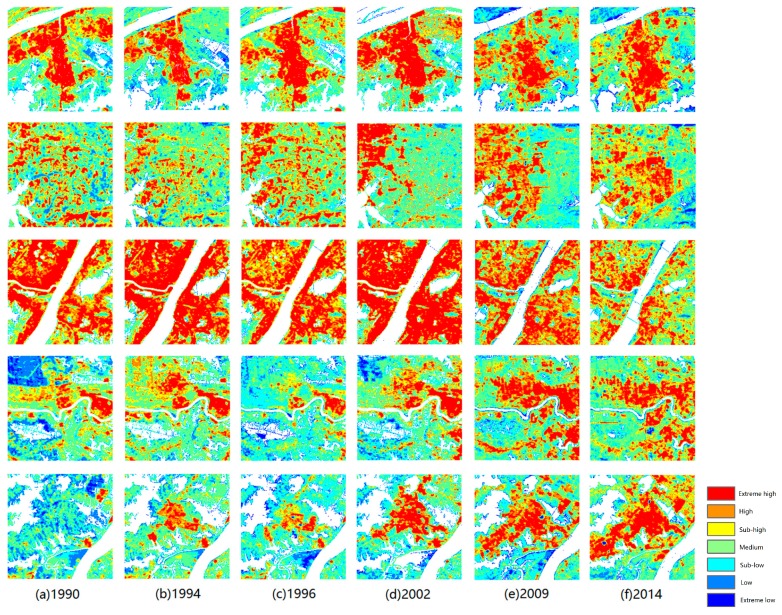
Surface urban heat island (SUHI) evolution (from top to bottom, circle 1 to circle 5; from left to right, the years 1990 to 2014).

Time-series tracking was used to analyze the variations in the five aggregations of SUHIs defined in the previous section, and it is illustrated in Figure 6: from top to bottom, circle 1 to circle 5 and from left to right, the years 1990 to 2014.

For the Wuhan Iron and Steel Group, the red areas that represent extreme high temperature regions extended before 2002 and were then broken down step by step. The same phenomenon appeared for the central downtown residential area, as shown in the third line of Figure 6. These extreme high temperature regions changed to high or sub-high regions.

For the East Lake High-Tech Development Zone, the red areas obviously became stronger. The red areas were symmetrically distributed from 1990 to 1996 when the development zone had not been constructed. After 2002, gradual development occurred from west to east. The Lin Kong Harbor Economic Technological Development Zone showed a nearly equivalent trend, except development occurred from the opposite direction. Additionally, a comparison of the 2009 and 2014 images showed that the aggregation red area was gradually disrupted.

The changes in the Zhuankou Economic Technological Development Zone were unique, and the extreme high temperature area expanded and strengthened from 1990 to 2014 and did not experience the disruption in the red area that occurred in other zones. The red areas grew year by year and showed considerable aggregation.

**Figure 7:**
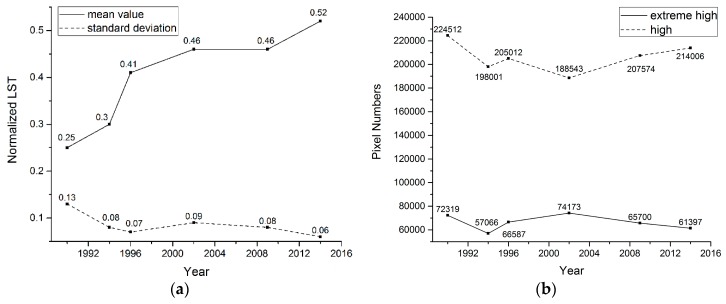
Statistic of (**a**) normalized LST; (**b**) pixel numbers of extreme high and above-high regions.

We calculated the normalized LSTs in the experiment to quantitatively evaluate the variation of SUHIs. Then, the statistics of the normalized LST and the pixel numbers of the extreme high and high regions were calculated. The results are shown in Figure 7. 

The mean value of the normalized LST increased from 1990 to 2014, revealing that the temperature increased overall in the Wuhan City Development area. However, the standard deviation stayed relatively steady (although it was slightly higher in 1990), which indicates that the overall LST slowly increased, and the gap between high LSTs and low LSTs did not increase. Changes in the pixel numbers indicated a similar situation. After 2002, the pixel numbers for the extreme high temperature region decreased, whereas the pixel numbers for high and above-high temperature regions increased. The extreme high region was then disrupted and changed to high or sub-high regions.

### 3.4. Relationship of the LCF to SUHI Intensity

SUHI intensity, which is defined as LST difference between the mean urban LST and the mean rural LST, is used as the classical indicator to quantitatively describe the SUHI effect [30]. The physical mechanism for the UHI is very complicated and is related to each component of energy balance due to the land use change [29,54]. One of the important factors that cause the SUHI effect is artificial heat storage objects, such as buildings and roads, which replace vegetation or lakes. Therefore, the different combinations of LCFs affect the SUHI intensity changes. Following one previously published study [55], our experiment used Origin software to draw the ternary triangle contour graphic of the endmember fractions with normalized LSTs. This graphic can accurately describe the relationship between 3D data and other thematic information.

**Figure 8:**
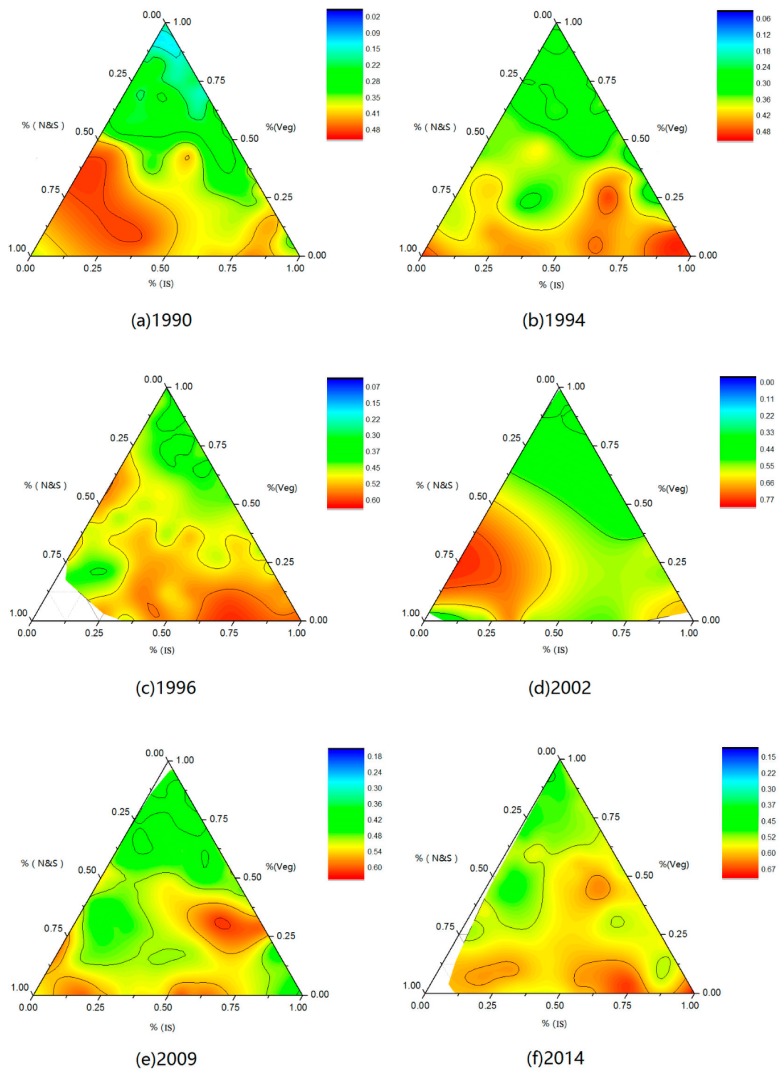
Ternary triangle contour graphics of endmember fraction with normalized LST.

The three sides of the triangle shown in Figure 8 are used to represent the fractions of IS, VEG, and N&S, and the depth of the color in the triangle represents the normalized LSTs, which represent the intensity of the SUHI. Note that the legend for each graph is not the same because we found that a well-distinguished interval had a better visual effect. Although the values of the corresponding colors are slightly different for each graphic, they reflect relatively high and low temperatures. 

The SUHI intensity is lowest for the top of the triangle; the VEG fraction is close to 1, and the other endmember fraction is close to 0. This trend is most prominent in the 1990 graphic. After 1990, the trend is not as obvious. By 2014, the color at the top of the triangle is lighter than that at the bottom, although it remains significantly warm. 

The warm (orange and red) areas are mostly concentrated in the bottom of the image, especially in the lower right corner of the triangle, which indicates that the fraction of IS is close to 1, and the SUHI effect has become more serious. 

From an overall perspective, the gaps between the colors gradually diminish from 1990 to 2014; thus, the experimental areas with differences in high temperatures and low temperatures are gradually reduced. Moreover, the hottest zones can be observed on the side of the triangle; thus, the maximum normalized LST occurs with only one endmember, usually the IS endmember or the N&S endmember.

### 3.5. Fitting Analysis on the SUHI Fierce Change Area

**Figure 9:**
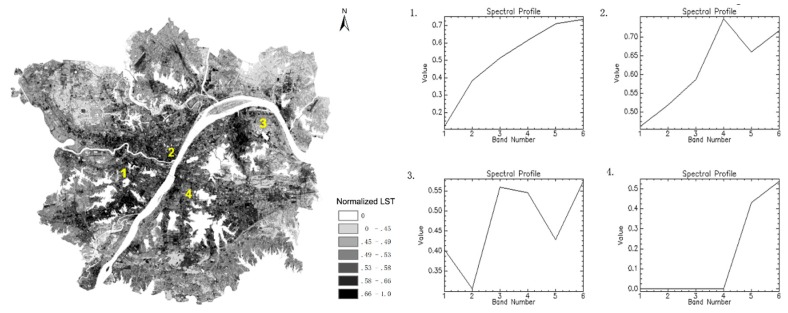
Normalized LST in 2014 with a sketch map of four defined situations.

In our experiment, layers were stacked and time trajectories were constructed for the six normalized LST images. The values with images are similar to the reflectance with bands. Connecting the values of each pixel produced spectra curves that reflected changes in the normalized LST with time. To explore the SUHI intensity variations for each pixel, we focused on these spectra curves and summarized their characteristics below. Four scenarios were thus defined as shown in Figure 9.

In the first scenario, a monotonously increasing spectral curve was observed. In areas that correspond to these curves, which were mainly distributed in the new development city, the normalized LST increased over time. In the second scenario, the curve declined over a certain time period and then rose over another period. This situation was the most widely distributed and generally occurred in the old city, although it was also observed in new urban areas. This curve trend was likely to occur in areas where the extreme high temperature region disintegrated. In the third scenario, the curve changes were not regular but rose and fell. This situation was mainly distributed in rural areas, especially in farmland due to crop changes. The normalized LST was lower in dense vegetation covers and higher in the presence of bare soil. In the fourth scenario, the beginning of the curve value was 0, and it then rose rapidly for a certain time period. This situation was unique and mainly distributed around lakes and rivers. The initial value of the curve was 0 because the temperature value was cut off by the water mask; however, the area was converted from a lake to land, and the normalized LST increased rapidly.

In this experiment, we defined the first scenario as an SUHI intensity fierce change area, which indicates that a rapid increase in SUHI intensity occurred in these areas. Moreover, we extracted the pixels that included the large change area and showed change gaps larger than 0.6 in the six images. These areas were the most obviously increasing SUHI zones in the experimental area. Then, a fitting analysis was performed between the SUHI intensity and the fractional information in the extracted pixels. The results are shown in Table 5.

In Table 5, IS, VEG and N&S represent the three-endmember fractions as previously noted. The results show that the three fractions could reflect the influence on the SUHI intensity. The IS fraction had the most positive effect on the SUHI followed by the N&S fraction. The VEG fraction coefficient could alleviate the occurrence of SUHIs. These results reveal the impact of the LCFs on SUHIs. Urban expansion generates impervious artificial objects that replace pervious natural objects, which causes an increase in LST and results in a SUHI.

## 4. Discussion

### 4.1. Time-Series Images in SUHI Research

The innovation of this research was the use of long-term satellite remote sensing data to monitor SUHIs. Many studies have used 1 or 2 years of remote sensing data to retrieve LSTs [25,26,27,28,31,32,33]. To determine variations of SUHIs, long-term remote sensing data are important. In this study, five Landsat-5 images and one Landsat-8 image were chosen. However, our original intention was to identify as many appropriate Landsat datasets as possible for a specific period; however, this attempt failed because of issues with data quality. Additionally, multi-sensor data were not easily used in this experiment. The different properties of the sensors, such as the radiation resolution and viewing angle, may also pose considerable challenges [35]. A suitable scale must be established to eliminate these differences. 

Several steps were carefully considered for the use of long-term images. We resized all images, including the thermal infrared band, to a resolution of 60 m to guarantee the same spatial resolution. Two methods were used for retrieving LSTs that fit the different data and provided more reliable results. Three categories of endmembers that match the exact ground features were defined for the endmember extraction step in the MESMA method. To eliminate the atmospheric effects, the endmembers were individually extracted. All these steps were performed to resolve the problems of inconsistency in the time-series images.

### 4.2. Mapping Methods for LCF and Normalized LST

A mapping method was used to describe the relationship between fractional data and normalized LST information, and it provided an intuitive reflection of the impact of land surface fraction combinations on normalized LSTs. However, different normalized LST distributions were observed because of the actual conditions of the images, and poor visual effects could have been produced if we had used the same legend bar for all six ternary triangle contour graphics. Therefore, we established different well-distinguished intervals so that each map could reflect the relatively high and low temperatures, although the values of the corresponding colors were slightly different.

A possible improvement to this method would be to parameterize the ternary triangle contour graphics. In our research, all the analyses were qualitative. In future studies, the centroids of triangles should be defined to quantitatively determine changes in the fraction combinations.

### 4.3. Fitting Analysis of the Specific Area

Our research analyzed the normalized temperature image spectra curves of the time series. Then, the large change areas were extracted based on the changing characteristics. A fitting analysis of the LCFs with the normalized LST was performed in the high-intensity SUHI change areas because a number of researchers have performed similar work for entire experimental areas [28,31,32,33,34,35,36]. However, we believe that our experiment was more suitable for three reasons. First, an entire experimental area with an enormous number of pixels may have led to information redundancy. Second, the thermal infrared bands, which had an original resolution of 120 m/100 m, were resized to 60 m, which caused a decrease in the LST retrieval accuracy. Therefore, different fraction combinations may have had the same LST, which would have confused the fitting analysis. Third, focusing on the SUHI large change areas can provide a better explanation of the increases in the normalized LSTs in these areas since 1990.

Additionally, instead of using only the pixels of one image for the fitting analysis, we used the pixels of large change areas in all six images. This process made good use of the time-series images and provided a convincing result.

## 5. Conclusions 

In this research, a series of Landsat satellite images was used to explore the relationship between SUHI and LCF changes in the Metropolitan Development Area of Wuhan in September and October from 1990 to 2014. The generalized single-channel method and the radiation transport equation method were used to obtain the LSTs and produce a thematic thermal map. We analyzed the distribution of and variations in the SUHI in the experimental area. We acquired LCF information by performing MESMAs, and then six ternary triangle contour graphics were drawn to explore the impact of endmember fraction combinations on the SUHI intensity. Finally, we summarized the changes in the characteristics of time-series-normalized LSTs and extracted the SUHI large change area. A linear fitting analysis was performed for this area in six images, and we found that impervious surfaces had the largest positive impacts on SUHI intensity followed by the non-chlorophyllous vegetation and soil fraction. In addition, the results showed that the green vegetation fraction could alleviate the occurrence of SUHIs. These results also reveal the impact of the land cover fractions on surface urban heat islands. Urban expansion generates impervious artificial objects that replace pervious natural objects, which causes an increase in land surface temperature and results in a surface urban heat island. Still, there remains some work to be conducted in the future such as a detailed inter-annual variation analysis based on more data of the three land cover fractions, and the statistical analysis of the SUHI variation during these 24 years.

## Figures and Tables

**Table 1:** Landsat images used in this study.

Satellite	Orbit	Time
Landsat 5	P123r039	1990/09/02
		1994/09/29
		1996/10/04
		2002/09/03
		2009/09/06
Landsat 8	P123r039	2014/10/06

**Table 2:** Definition of land surface temperature region. LST: land surface temperature; LST mean; average LST; STD: standard deviation.

Region	Definition
extreme low	LST < LST mean − 1.5*STD
low	LST mean − 1.5*STD < LST < LST mean − STD
sub-low	LST mean − STD < LST < LST mean − 0.5*STD
medium	LST mean − 0.5*STD < LST < LST mean + 0.5*STD
sub-high	LST mean + 0.5*STD < LST < LST mean + STD
high	LST mean + STD < LST < LST mean + 1.5*STD
extreme high	LST > LST mean + 1.5*STD

**Table 3:** Area of the three endmembers in each year.

Categories of Endmembers	Area (km^2^)					
	1990	1994	1996	2002	2009	2014
IS	144.30	166.29	227.27	166.94	186.04	128.42
VEG	395.30	361.94	310.95	360.01	319.95	379.24
N&S	33.83	76.65	82.66	18.76	109.01	75.69

IS: impervious surfaces; VEG: green vegetation; N&S: non-chlorophyllous vegetation and soil.

**Table 4:** Confusion matrix, overall accuracy and Kappa coefficient.

**Year**	**Category**	**IS**	**VEG**	**N&S**	**Total**	**Year**	**Category**	**IS**	**VEG**	**N&S**	**Total**
1990	IS	72	0	11	83	1994	IS	86	0	8	94
	VEG	0	71	13	84		VEG	0	91	36	127
	N&S	3	0	47	50		N&S	7	2	51	60
	Total	75	71	71	217		Total	93	93	95	281
	Overall Accuracy = (190/217) = 87.56%						Overall Accuracy = (228/281) = 81.14%				
	Kappa Coefficient = 0.81						Kappa Coefficient = 0.72				
**Year**	**Category**	**IS**	**VEG**	**N&S**	**Total**	**Year**	**Category**	**IS**	**VEG**	**N&S**	**Total**
1996	IS	80	6	14	100	2002	IS	54	1	16	71
	VEG	4	87	16	107		VEG	3	90	10	103
	N&S	8	0	53	61		N&S	2	0	55	57
	Total	92	93	83	268		Total	59	91	81	231
	Overall Accuracy = (220/268) = 82.09%						Overall Accuracy = (199/231) = 86.15%				
	Kappa Coefficient = 0.73						Kappa Coefficient = 0.79				
**Year**	**Category**	**IS**	**VEG**	**N&S**	**Total**	**Year**	**Category**	**IS**	**VEG**	**N&S**	**Total**
2009	IS	68	13	8	89	2014	IS	70	14	27	111
	VEG	1	85	9	95		VEG	1	85	13	99
	N&S	27	0	77	104		N&S	10	0	42	52
	Total	96	98	94	288		Total	81	99	82	262
	Overall Accuracy = (230/288) = 79.86%						Overall Accuracy = (197/262) = 75.19%				
	Kappa Coefficient = 0.70						Kappa Coefficient = 0.63				

**Table 5:** Results of the fitting analysis.

Categories of Endmembers	Coefficient	*F*-Value	*P*-Value	Adjust R^2^
Intercept	0.355	231.63796	0	0.454
IS	0.421			
VEG	−0.191			
N&S	0.203			

## Abbreviations

The following abbreviations are used in this manuscript:

SUHI	surface urban heat island
LULC	land use and land cover
NDVI	normalized difference vegetation index
LCF	land cover fraction
LST	land surface temperature
MESMA	multiple endmember spectral mixture analysis
MNDWI	modified normalized difference water index
